# Increased Oxidative Stress and Inflammation Independent of Body Adiposity in Diabetic and Nondiabetic Controls in* falciparum* Malaria

**DOI:** 10.1155/2016/5216913

**Published:** 2016-05-19

**Authors:** Samuel Acquah, Johnson Nyarko Boampong, Benjamin Ackon Eghan Jnr

**Affiliations:** ^1^Department of Medical Biochemistry, School of Medical Sciences, College of Health and Allied Sciences, University of Cape Coast, Cape Coast, Ghana; ^2^Department of Biomedical and Forensic Sciences, School of Biological Sciences, University of Cape Coast, Cape Coast, Ghana; ^3^Department of Medicine, School of Medical Sciences, Kwame Nkrumah University of Science and Technology, Kumasi, Ghana

## Abstract

Information on the extent to which oxidative stress and inflammation occur in the presence of* falciparum* malaria and type 2 diabetes mellitus in the same individual is limited. This study sought to investigate the extent of inflammation and oxidative stress in adult uncomplicated malaria by measuring fasting levels of lipid peroxides, C-reactive protein (CRP), and total antioxidant power (TAP) before and during* falciparum* malaria, in 100 respondents with type 2 diabetes and 100 age-matched controls in the Cape Coast metropolis of Ghana. Also, body adiposity index, body mass index, and waist-to-hip ratio were computed. Before and during* falciparum* malaria, diabetes patients exhibited higher (*P* < 0.05) levels of CRP and peroxides than controls but TAP and BAI were comparable (*P* > 0.05) between the two groups. Baseline CRP correlated positively (*r* = 0.341, *P* = 0.002) with peroxide only in the diabetic group. During malaria, TAP level in both study groups declined (*P* < 0.05) by 80% of their baseline levels. CRP correlated negatively (*r* = −0.352, *P* = 0.011) with TAP in the control but not the diabetic group. Uncomplicated* falciparum* malaria elevated inflammation and peroxidation but decreased antioxidant power independent of adiposity. This finding may have implication on cardiovascular health.

## 1. Introduction

Malaria and type 2 diabetes mellitus (T2DM) continue to afflict millions of people despite aggressive efforts at controlling them. With malaria declining globally, T2DM is on the rise with developing countries predicted to shoulder majority of the burden [1, 2]. While T2DM is a metabolic disorder characterised by high blood glucose level, malaria is a parasitic disease caused by the* Plasmodium* parasite. In spite of the seeming variations between the two conditions, they appear to adopt similar mechanisms in perpetrating their negative health effects on their host. Three interrelated mechanisms common to the pathogenesis of both T2DM and malaria include inflammation, oxidative stress, and reduced antioxidant capacity.

Inflammation is considered as a normal immune mechanism employed by humans and other animals to protect against attack by pathogens and other foreign materials. However, in recent times, inflammation has been found to distort cellular integrity and function, leading to several diseases and risk factors such as insulin resistance, obesity, T2DM, and other cardiovascular-related chronic conditions [3–6].

Currently, malaria is viewed as an inflammatory cytokine-driven condition with* Plasmodium falciparum* malaria considered as the single most daring form of human malaria with the greatest human suffering in modern times [7, 8]. Various inflammatory mediators such as C-reactive protein (CRP) and tumour necrosis factor-*α* (TNF-*α*) have been found to increase in levels with increased* P. falciparum* parasitaemia, possibly due to increased expression levels [9–11]. In Ghana,* falciparum* malaria continues to be the major cause of morbidity and out-patient visit to health facilities.

Inflammation and oxidative stress are strongly related and each can be caused by the other. For instance, the protective role of inflammation in preventing the survival and growth of pathogens is mediated through oxidative stress. On the other hand, extensive release of markers of oxidative stress modifies cellular targets in a manner that signals inflammatory response. Oxidative stress is a complex process that results from imbalance between reductive and oxidative capacities of cells. It is caused by heterogeneous group of compounds called reactive oxygen/nitrogen species (ROS/RNS) [12]. Apart from pathogens, ROS/RNS can be generated from mitochondria-dependent breakdown of fuel molecules to generate energy for various cellular activities. The heterogeneous nature of ROS/RNS gives rise to a variety of oxidative products that can be assayed in a given health condition. ROS/RNS usually execute their detrimental effect through generation of highly unstable and reactive free radicals that perpetuate chain reaction mechanism by abstracting unpaired electrons from other molecules to destabilize them [12]. In this regard ROS/RNS can affect every cellular component. Biochemically, oxidative stress can be assessed in serum by measuring levels of 2-thiobarbituric acid reactive substances (TBARS) which is generally reported in malondialdehyde (MDA) equivalent.

Interestingly, the exact effect of ROS/RNS depends on the availability and activity of cellular antioxidant compounds. Cellular antioxidants systems work to prevent the formation of new ROS/RNS, halt the radical chain reaction by removing ROS/RNS when they are formed, or make them harmless [12]. Several compounds and enzyme systems function as antioxidants, making it difficult to assay them individually in any given research. Biochemically, the relevance of any biomolecule is determined by the net effects of its interactions with other molecules in the cell. Therefore, the interactions among the various antioxidants systems and with other cellular components should determine the overall antioxidants status of the cell, referred to as total antioxidant power/capacity [13].

Although the levels of inflammation, oxidative stress, and antioxidants have been acknowledged extensively in T2DM and malaria separately, information on the extent to which they occur in the presence of both* falciparum* malaria and T2DM in the same individual is limited. Therefore, the current study seeks to examine changes in levels of CRP, lipid peroxides, and total antioxidant power (TAP) due to* falciparum* malaria in the same individual with T2DM compared with age-matched nondiabetic controls, over a two-year period, in the Cape Coast metropolis of Ghana.

## 2. Materials and Methods

### 2.1. Study Site, Selection of Participants, and Sample Collection

#### 2.1.1. Study Site

The study was conducted at the Cape Coast Teaching Hospital (CCTH) which serves as the referral hospital in the Central Region of Ghana. CCTH has a well-structured diabetes clinic that manages diabetics in the metropolis and the region at large. The characteristics of patients who utilise facilities at CCTH reflect those of the entire region. The region has two ecological zones: the forest and the coastal zones with a typical tropical weather influenced by South West Monsoon winds (March–October) and North East Trade winds (November–February). The temperatures average between 21 and 32°C throughout the year with an annual rainfall value of 750–1000 mm. The inhabitants of the region are mostly farmers and fishermen in the informal sector of the economy. A relatively smaller fraction of the inhabitants are in the formal sector of the economy. Cape Coast, the capital of the region, is home to a number of educational institutions in the region.

#### 2.1.2. Selection of Participants, Anthropometry, and Sample Collection for Laboratory Analyses

The study involved 100 respondents with diabetes and 100 age-matched nondiabetic controls aged 32–80 years. Respondents with diabetes were randomly selected from a pool of diabetes patients attending the diabetic clinic at CCTH with the controls from the general inhabitants of the Cape Coast metropolis. All participants were physically examined by a medical team and screened appropriately before enrollment on the study. Ten-millimeter blood sample was taken from each participant after an overnight fast before and during* falciparum* malaria. Blood samples were separated into plasma and serum for various biochemical analyses. Details on participants' selection, sample preparation, and storage have been published elsewhere [14].

Height was measured to the nearest 0.1 cm with hip circumference measured around the broadest portion of the buttocks. Body adiposity index (BAI) was computed in accordance with the formula developed by Bergman et al. [15]. BAI is a new index thought to be superior to BMI in measuring body adiposity. Above all, weight and waist circumference were measured following standard protocol for subsequent computation of body mass index (BMI) and waist-to-hip ratio.

### 2.2. Laboratory Analyses

#### 2.2.1. Determination of C-Reactive Protein (CRP)

Measurement of serum CRP was done by commercially available sandwich enzyme-linked immunosorbent assay (ELISA) kits procured from Assaypro, USA (Assaypro Inc., USA). The assay procedure was in accordance with manufacturer's instruction. The detection limit of the assay was 0.01 ng/mL and the intra-assay and interassay coefficients of variation were 5.4% and 7.7%, respectively.

#### 2.2.2. Determination of Total Antioxidant Power and Lipid Peroxides

Total antioxidant power (TAP) and lipid peroxides in serum samples were assayed by kits procured from Oxford Biomedical (Oxford Biomedical Research, USA), following the given instructions. TAP was measured as trolox equivalent in mM by a microplate-based colorimetric assay that relies on the reducing ability of copper (II) ion to copper (I) ion. However, lipid peroxidation products in serum samples were determined spectrophotometrically as 2-thiobarbituric acid reactive substances (TBARS) reported generally in malondialdehyde (MDA) equivalent.

#### 2.2.3. Diagnosis of* Falciparum* Malaria and Diabetes

*Falciparum* malaria was diagnosed by the presence of appropriate clinical signs and symptoms together with a positive rapid diagnostic test for* P. falciparum* in blood. The CareStart*™* Malaria HRP2Pf rapid diagnostic test kit (Access Bio Inc., USA), which has been extensively evaluated and found to be highly sensitive and specific for* P. falciparum* and with strong correlation to microscopy [16] was used. Type 2 diabetics were already diagnosed diabetic patients who were receiving treatment at CCTH.

### 2.3. Ethical Approval

Ethical approval for the study was granted by the Committee on Human Research, Publications and Ethics of the Kwame Nkrumah University of Science and Technology, Kumasi. All protocols used in conducting the study were in line with the ethical standards of the CCTH, Ghana Health Service, and the World Medical Association declaration of Helsinki. Above all, written informed consent was obtained from all study participants.

### 2.4. Statistical Analysis

Data were analyzed by Statistical Package for Social Sciences (SPSS) software version 17 and presented as mean ± standard deviation. The two-tailed independent sample *t*-test was used to compare mean levels of parameters between study groups and gender, where appropriate, before and during malaria. Pearson correlation, linear stepwise, and logistic regression analyses were performed. A *P* value < 0.05 was considered statistically significant.

## 3. Results

The study involved a total of 200 respondents (100 diabetics and 100 nondiabetic controls) of which 100 (70 diabetics and 30 nondiabetic controls) had malaria during a two-year follow-up period. At baseline, respondents with diabetes exhibited higher (*P* < 0.05) levels of CRP, peroxides, BMI, and WC but lower WHR and comparable (*P* > 0.05) level of TAP and BAI than their nondiabetic counterpart (Table 1). Analysis of data in each study group by gender did not reveal any gender-specific variation in levels of any of the measured biomarkers but higher BMI, WHR, and WC in diabetic females only (data not shown). A similar analysis of data between groups showed that males with diabetes had higher (*P* < 0.05) levels of peroxides and CRP but lower WHR than their control counterpart (Table 1). In the females, respondents with diabetes exhibited higher (*P* < 0.05) BMI, WC, CRP, and peroxides than their nondiabetic controls (Table 1).

In the presence of* falciparum* malaria, respondents with diabetes maintained their superiority (*P* < 0.05) over their nondiabetic counterpart in terms of levels of CRP, peroxides, BMI, WHR, and WC but comparable (*P* > 0.05) TAP and BAI levels (Table 2).

**Figure 1:**
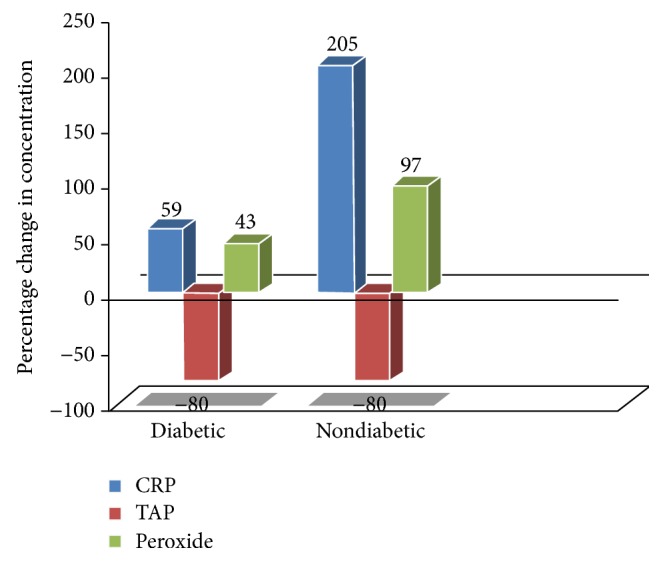
Percentage change in concentration of biomarkers due to* falciparum* malaria.

Compared to mean baseline levels of the measured parameters, a statistically significant (*P* < 0.05) increase in levels of CRP and peroxides and decrease in TAP level were observed in both study groups during malaria without any appreciable change in level of any of the indices of adiposity (data not shown). Interestingly, the two groups differed in their extent of elevation of CRP and peroxides but their extent of decrease in TAP levels during malaria was equal (Figure 1).

Apart from baseline CRP level which correlated positively (*r* = 0.341, *P* = 0.002) with peroxide level in the diabetic group, no correlation (*P* > 0.05) was observed between any of the measured biomarkers in the study groups. During malaria, a negative correlation (*r* = −0.352, *P* = 0.011) between CRP and TAP was rather found in the control group only. The weak correlation in each study group could not be confirmed (*P* > 0.05) in subsequent study group-based stepwise regression analyses. However, when the data in the two study groups were pooled together and treated as one, the stepwise regression analyses showed that malaria-induced CRP level could be independently (*R* ^2^ = 0.749 and adjusted *R* ^2^ = 0.591; *P* = 0.026) predicted by baseline CRP level only. The model could explain about 59% of the observed variation in CRP levels of respondents who had* falciparum* malaria.

A multiple logistic regression analysis with malaria as the dependent variable and changes in levels of CRP, TAP, peroxides, and anthropometric indices as the independent variables revealed a significant (*P* < 0.05) association between* falciparum* malaria and changes in levels of CRP, TAP, and lipid peroxides independent of the anthropometric indices after adjusting for age, diabetes status, and other baseline indices (Table 3).

## 4. Discussion

Measures of inflammation, peroxidation, and antioxidants status can be used as valuable indicators for assessment of disease risk, severity or progression, and treatment outcomes. In the current study, CRP, lipid peroxides, and TAP were measured as markers of inflammation, peroxidation, and antioxidants status, respectively. Although inflammation has been studied in several disease conditions through the use of varied inflammatory markers due probably to the complex nature of the phenomenon, CRP, an acute phase reactant protein, stands out as the most dependable and extensively studied marker for assessing cardiovascular disease risk, hence its use in the current study [17]. The elevated trend of baseline CRP level for diabetics compared with controls in the current study is in support of a number of earlier studies [18, 19]. However, the mean level of CRP in the current study is higher than the earlier reports [18, 19]. This observation could be ascribed to variations in characteristics of respondents in the current study such as sample size and race compared to the previous ones [18, 19]. Indeed, Africans have been reported to have a unique mutation in the CRP gene that results in elevated circulating levels than their Caucasian counterpart [20]. Although elevated CRP level of Africans is viewed as a beneficial factor of protection against infectious diseases, it could indicate increased risk to cardiovascular disease as well as other inflammation-dependent health conditions [21]. In the presence of* falciparum* malaria, CRP levels increased in both study groups, corroborating several previous reports [9, 11, 21]. The extent of increase in CRP level was higher for the control than the diabetic group, suggesting that control respondents in the current study, probably, had relatively higher* falciparum* malaria parasitaemia than their diabetic counterpart. Despite the fact that the qualitative RDT used for malaria diagnosis in the current study could not determine degree of infection directly, a number of earlier reports have observed that CRP levels increase with increased* falciparum* parasitaemia [9, 11]. To this end, a change in CRP level could be a reflection of a corresponding change in level of* falciparum* parasitaemia as seen in the present study. This finding also suggests that the perceived protection of Africans to infection due to genetically induced elevation of CRP may not be sustainable at all times. In the present study, diabetics with higher baseline CRP seem to have increased vulnerability to* Plasmodium falciparum* infection as reported previously [22]. Considering the fact that individuals with asymptomatic* Plasmodium falciparum* infection exhibit low circulating CRP levels, the elevated level of CRP in* falciparum* malaria patients in the current study should not be seen as an ordinary acute phase response [21, 23]. Rather, it could be a reflection of an increased risk of cardiovascular event, particularly, in high-risk individuals such as diabetics in the current study. With recent evidence linking inflammation to various cardiovascular-related health conditions, the elevated CRP in malaria, observed for even the nondiabetic control respondents of the present study, could be indicative of increased risk of cardiovascular event, particularly, when the possibility of multiple episodes of symptomatic* Plasmodium falciparum* infection is high in the Ghanaian setting [9, 11].

Diabetics exhibited higher serum peroxidation levels expressed as malondialdehyde (MDA) before* falciparum* malaria. This baseline observation supports numerous reports and confirms the central role of oxidative stress in diabetes pathogenesis [24, 25]. Indeed, the hyperglycaemia, which is a characteristic feature of diabetes, activates several biochemical pathways that ultimately give rise to products that cause oxidative stress. At baseline, level of peroxidation correlated positively with CRP in the diabetic group suggesting a synergistic relationship between inflammation and peroxidation in this study group. In a number of longitudinal studies involving relatively large number of respondents, serum lipid peroxidation could independently predict cardiovascular events in patients with stable coronary heart disease or diabetes [26, 27]. These reports together with the observation in the present study point to an increased risk of cardiovascular event in the diabetic group compared to the controls.

As expected, MDA levels increased in both study groups in* falciparum* malaria in line with several reports from both human and animal studies [28, 29]. Indeed, enhanced peroxidation, is an immune defense mechanism adopted by host cells to get rid of invading pathogens. Nonetheless, the process could be detrimental to the host as well. The degree of peroxidation observed during* falciparum* malaria was higher in controls than the diabetics group, reiterating the likelihood that the controls had a relatively higher parasitaemia. Since the overall impact of peroxidation reaction depends on the action of the antioxidant status of the cell, we investigated total antioxidant power (TAP) also called total antioxidant capacity in relation to malaria in the two study groups. Interestingly, the two groups exhibited comparable TAP levels at baseline indicating that the enhanced levels of inflammation and peroxidation observed in diabetics than controls was not driven by varied TAP levels in the two groups of respondents. This trend is at variance with several works that reported higher and lower antioxidant levels, respectively, for controls and diabetics but in support of Taheri et al. [26, 30, 31]. Although the observed variation between the current work and the earlier ones could be ascribed to variation in sample size, age, degree of central obesity, and the apparent health condition of control respondents used in the studies, the sensitive nature of TAP to other environmental factors such as diet, exercise, and medication cannot be overlooked [32]. As expected, TAP levels plummeted in both groups during malaria suggesting that* falciparum* malaria seems to have interfered with the antioxidant system of respondents to reduce their capacity to prevent free-radical-dependent oxidation as previously reported [33]. This observation makes the need for inclusion of antioxidants in malaria treatment attractive.

Adiposity has long been associated positively with inflammation and oxidative stress in several reports involving respondents of varying health status, age, race, and index of adiposity [15, 34–36]. The adipose tissue secretes several inflammatory cytokines with associated oxidative stress which links it to the several reported health consequences [3–8]. Recently, the BAI which was thought to be superior to BMI in measuring adiposity [16] did not differ between gender or study groups in our respondents. Also, conventional measures of adiposity such as BMI, WC, and WHR did not associate with any of the measured biomarkers, suggesting that the observed variations in inflammatory and oxidative stress markers during malaria were independent of adiposity as indicated by the logistic regression model. This observation is at variance with previous reports [15, 34–36] that associated markers of inflammation and oxidative stress with adiposity. The observed variation between the current study and the previous ones [15, 34–36] could be due to differences in sample size, age, and health status of respondents and the specific index of adiposity used. Indeed, the superiority of BAI over BMI and other measures of adiposity is gender-dependent [37], as statistical control of gender attenuates the strength of BAI to better predict adiposity compared with conventional indices.

Considering the central role of oxidative stress and inflammation in cardiovascular health independent of adiposity, our current findings appear to support increased risk of respondents to cardiovascular event in the presence of* falciparum* malaria.

## 5. Conclusion

Uncomplicated* falciparum* malaria increased levels of inflammation and oxidative stress markers in diabetics and nondiabetic controls independent of adiposity. These findings may have implications for cardiovascular health of respondents.

## Figures and Tables

**Table 1:** Baseline CRP, TAP, and peroxide levels of respondents by study group and gender.

Parameter	Diabetic *N* = 100	Nondiabetic *N* = 100	*P* value
CRP (mg/L)	3.75 ± 0.33	1.54 ± 0.47	<0.001^*∗*^
TAP (*μ*M)	226.36 ± 26.40	251.71 ± 18.30	0.416
Peroxide (*μ*M)	16.82 ± 2.01	9.91 ± 1.70	0.01^*∗*^
BAI	31.90 ± 6.06	32.77 ± 6.06	0.464
BMI (kg/m^2^)	28.37 ± 6.65	25.23 ± 5.34	0.022^*∗*^
WC (cm)	97.27 ± 1.14	92.00 ± 1.13	0.003^*∗*^
WHR	0.91 ± 0.10	0.96 ± 0.11	<0.001^*∗*^

Male respondents			
	*N* = 32	*N* = 26	

CRP (mg/L)	2.77 ± 0.37	1.07 ± 0.48	0.02^*∗*^
TAP (*μ*M)	206.68 ± 32.60	233.13 ± 20.10	0.669
Peroxide (*μ*M)	16.46 ± 1.80	9.46 ± 1.80	0.007^*∗*^
BAI	32.06 ± 6.36	33.62 ± 8.95	0.455
BMI (kg/m^2^)	25.72 ± 1.41	24.74 ± 1.23	0.488
WC (cm)	91.69 ± 11.24	89.33 ± 10.92	0.409
WHR	0.86 ± 0.11	0.98 ± 0.15	<0.001^*∗*^

Female respondents			
	*N* = 68	*N* = 74	

CRP (mg/L)	2.78 ± 0.37	1.83 ± 0.46	0.001^*∗*^
TAP (*μ*M)	206.68 ± 32.60	260.08 ± 17.50	0.533
Peroxide (*μ*M)	16.46 ± 1.80	9.45 ± 2.0	0.009^*∗*^
BAI	31.90 ± 6.06	32.77 ± 6.06	0.464
BMI (kg/m^2^)	31.01 ± 1.5	25.71 ± 1.23	<0.001^*∗*^
WC (cm)	102.85 ± 1.13	93.86 ± 1.14	<0.001^*∗*^
WHR	0.94 ± 0.20	0.93 ± 0.16	0.37

Figures represent mean ± standard deviation; CRP = C-reactive protein; TAP = total antioxidants power; *∗* = significant *P* value; BAI = body adiposity index; BMI = body mass index; WC = waist circumference; WHR = waist-to-hip ratio.

**Table 2:** Levels of CRP, TAP, peroxides, and anthropometric indices during malaria.

Index	Diabetic (*N* = 70)	Nondiabetic (*N* = 30)	*P* value
CRP (mg/L)	5.95 ± 0.40	4.69 ± 0.36	0.037^*∗*^
TAP (*μ*M)	46.29 ± 13.02	49.76 ± 9.67	0.34
Peroxide (*μ*M)	24.01 ± 1.01	19.53 ± 1.96	0.026^*∗*^
BAI	32.10 ± 5.86	32.59 ± 5.91	0.73
BMI (kg/m^2^)	27.95 ± 6.95	24.89 ± 4.42	0.02^*∗*^
WC (cm)	96.58 ± 1.24	91.51 ± 1.38	0.011^*∗*^
WHR	0.89 ± 0.10	0.94 ± 0.14	<0.001^*∗*^

Figures represent mean ± standard deviation; CRP = C-reactive protein; TAP = total antioxidants power; *∗* = significant *P* value; BAI = body adiposity index; BMI = body mass index; WC = waist circumference; WHR = waist-to-hip ratio.

**Table 3:** Logistic regression analysis with malaria as the dependent variable and changes in CRP, TAP, and peroxides as predictors after adjusting for diabetes, age, and other baseline indices.

Parameter	*B*	SE	Wald	*P* value	Exp(*B*)
CRP	1.62	0.759	4.563	0.02^*∗*^	0.35
TAP	1.378	0.621	4.911	0.039^*∗*^	0.471
Peroxide	1.23	0.656	3.513	0.041^*∗*^	0.346
BMI	0.194	0.189	1.054	0.79	0.354
WHR	0.003	0.039	0.004	0.86	0.071
BAI	0.002	0.038	0.006	0.92	0.053
WC	0.003	0.080	0.001	0.99	0.23
Constant	−0.749	0.238	9.910	0.002	0.473

CRP = C-reactive protein; TAP = total antioxidants power; *∗* = significant *P* value; BAI = body adiposity index; BMI = body mass index; WC = waist circumference; SE = standard error.
